# CDKL3 shapes immunosuppressive tumor microenvironment and initiates autophagy in esophageal cancer

**DOI:** 10.3389/fimmu.2024.1295011

**Published:** 2024-03-18

**Authors:** Yanping Bi, Jie Liu, Songbing Qin, Fuqing Ji, Chao Zhou, Haihua Yang, Suna Zhou

**Affiliations:** ^1^ Department of Radiation Oncology, Xi’an No.3 Hospital, The Affiliated Hospital of Northwest University, Xi’an, Shaanxi, China; ^2^ Department of Medical Research Center, Xi’an No.3 Hospital, The Affiliated Hospital of Northwest University, Xi’an, Shaanxi, China; ^3^ Department of Radiation Oncology, The First Affiliated Hospital of Soochow University, Suzhou, Jiangsu, China; ^4^ Department of Thyroid Breast Surgery, Xi’an No.3 Hospital, The Affiliated Hospital of Northwest University, Xi’an, Shaanxi, China; ^5^ Department of Radiation Oncology, Taizhou Hospital Affiliated to Wenzhou Medical University, Taizhou, Zhejiang, China; ^6^ Key Laboratory of Minimally Invasive Techniques & Rapid Rehabilitation of Digestive System Tumor of Zhejiang Province, Taizhou, Zhejiang, China

**Keywords:** esophageal carcinoma, prognosis, tumor microenvironment, autophagy, macrophage polarization

## Abstract

**Background:**

CDKL3 has been associated with the prognosis of several tumors. However, the potential role of CDKL3 in immunotherapy and the tumor microenvironment (TME) in esophageal carcinoma (ESCA) remains unclear.

**Methods:**

In this study, Cox regression analysis was used to assess the predictive value of CDKL3 for ESCA outcomes. We systematically correlated CDKL3 with immunological features in the TME. The role of CDKL3 in predicting the efficacy of immunotherapy was also analyzed. Correlation analysis, Cox analysis and LASSO Cox regression were used to construct the CDKL3-related autophagy (CrA) risk score model. The relationship between CDKL3 expression and postoperative pathological complete response (pCR) rate in esophageal squamous cell carcinoma (ESCC) patients undergoing neoadjuvant chemoradiotherapy (nCRT) was evaluated using Immunohistochemical staining (IHC). The relationship between CDKL3 expression and autophagy induction was confirmed by immunofluorescence staining and western blot, and the effect of CDKL3 expression on macrophage polarization was verified by flow cytometry.

**Results:**

High expression of CDKL3 was found in ESCA and was associated with poor prognosis in ESCA. Moreover, CDKL3 expression was negatively correlated with tumor-infiltrating immune cells (TIICs), the integrality of the cancer immunity cycles, and anti-tumor signatures, while CDKL3 expression was positively correlated with suppressive TME-related chemokines and receptors, immune hyperprogressive genes, and suppressive immune checkpoint, resulting in immunosuppressive TME formation in ESCA. An analysis of immunotherapy cohorts of the ESCA and pan-cancer showed a better response to immunotherapy in tumor patients with lower CDKL3 levels. The CrA risk score model was constructed and validated to accurately predict the prognosis of ESCA. Notably, the CrA risk score of ESCA patients was significantly positively correlated with M2 macrophages. Furthermore, knockdown CDKL3 in KYSE150 cells could inhibit autophagy induction and M2 macrophage polarization. And, radiation could downregulate CDKL3 expression and autophagy induction, while ESCC patients with high CDKL3 expression had a significantly lower response rate after nCRT than those with low CDKL3 expression.

**Conclusion:**

CDKL3 may play an important role in anti-tumor immunity by regulating autophagy to promote the formation of immunosuppressive TME, thus playing a critical role in the prognosis of ESCA.

## Introduction

Esophageal carcinoma (ESCA) is a common malignancy affecting the gastrointestinal tract, with high incidence and mortality worldwide, of which 85% is esophageal squamous cell carcinoma (ESCC) (1, 2). ESCA typically does not present with early symptoms, resulting in the majority of patients being diagnosed in locally advanced or advanced stages. The primary treatment options for these locally advanced ESCA patients are neoadjuvant or definitive chemoradiotherapy (CRT), chemoradiation, or the combination of CRT and immunotherapy (3). The therapeutic efficacy of immunotherapy has continued to make breakthroughs in recent years, bringing light to the treatment of ESCA patients (4–7). Unlike conventional chemotherapy, immunotherapy can lead to unprecedented and durable remissions in advanced cancer patients. Unfortunately, only a subset of patients respond to immunotherapy, and clinical outcomes in ESCA patients vary widely (8). Therefore, the search for predictive biomarkers of immunotherapy benefits could help to personalize the treatment regimen for each patient and improve their prognosis.

The anti-tumor effects of immunotherapy require not only a tumor microenvironment (TME) with rich infiltration of immune cells but also active T cells by immune checkpoint inhibitors (ICIs) blocking immunosuppression (9, 10). Chemoradiotherapy can not only kill the fast-growing cancer cells, but it can also remodel the TME (11, 12). Autophagy, a mechanism of cellular self-protection and maintenance of homeostasis, removes senescent, damaged, or abnormal proteins and organelles from the cell (13). Aberrant activation of autophagy leads to tumor growth, endurance, and resistance to chemoradiotherapy. Radiotherapy is accompanied by abnormal expression of autophagy related-genes. Resistance to chemotherapy drugs is at least partially mediated by increased autophagy in tumor cells (14, 15). There is emerging evidence that autophagy causes immune dysfunction by acting on the TME. For example, TRAF2 promotes the polarization of M2 macrophages by inhibiting autophagy (16). Hence, exploring the features of the TME, the molecular features of autophagy and the interaction with chemoradiotherapy will help to understand the genesis and development of ESCA and the potential mechanisms of action of immunotherapy.

Members of the cyclin-dependent kinase (CDK) family regulate cell cycle progression and are considered crucial targets for cancer therapy (17). Cyclin-dependent kinase-like (CDKL) proteins contain MAPK TXY phosphorylation motifs, and putative cell cycle protein-binding domains and are characterized by their high sequence similarity to CDK. Cyclin-dependent kinase-like 3 (CDKL3) is both a protein-coding gene and a member of the CDKL family (18). Existing research demonstrates that tumor patients with CDKL3 up-regulation are closely related to inferior survival status (19–22). Our previous study identified CDKL3 as an important oncogene in esophageal squamous cell carcinoma (ESCC) and autophagy-related gene ATG5 was a potential target of CDKL3 in KYSE-150 cell line (19, 22). However, the effect of CDKL3 on TME and its role in autophagy are still unknown. Accordingly, our study aimed to investigate the association of CDKL3 with the TME and autophagy genes in ESCA based on public databases and experimental validation.

## Materials and methods

### Study design

**Figure 1:**
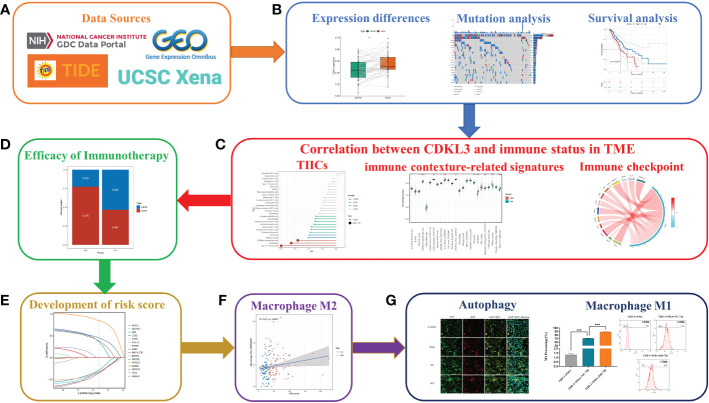
Flowchart of the study design. **(A)** Source of data used in this study. **(B)** Differential expression of CDKL3, mutation analysis, and survival analysis. **(C)** Correlation between CDKL3 and immune status in the TME. Immune status includes tumor-infiltrating immune cells (TIICs), immune-associated gene sets, and immune checkpoints. **(D)** The predicted role of CDKL3 in the efficacy of immunotherapy. **(E)** Establishing and validating CrA risk score model. **(F)** The association between the CrA risk score and M2 macrophages. **(G)** The correlation between CDKL3 expression and autophagy induction was verified by immunofluorescence staining, and the effect of CDKL3 expression on M1 macrophage polarization was verified by flow cytometry.

This study is performed according to the flow chart, which is shown in **Figure 1**. Firstly, data on ESCA patients were collected from public databases. Then, bioinformatics analysis was performed to explore the relationship between CDKL3 and immune status and the predictive role of CDKL3 in immunotherapy. Subsequently, the CDKL3-related autophagy (CrA) risk score model was developed and validated, and the correlation between the CrA risk score and M2 macrophages was found. Finally, through *in vitro* experiments, we confirmed the correlation between CDKL3 expression and autophagy induction and investigated the effect of CDKL3 on macrophage polarization.

### Data collection

We downloaded RNA sequencing (RNA-seq) data (transcripts per kilobase million, TPM values), mutation profiles, and clinical data of ESCA from TCGA-GDC interface (https://portal.gdc.cancer.gov/). Log2 was used to transform the RNA-seq data. 198 samples were included in the TCGA-ESCA sequencing data, of which 13 were normal tissues and 185 were tumor tissues. Somatic mutation information of ESCA patients from TCGA was plotted using the maftools R package. Copy number variation (CNV) data were accessed from the UCSC Xena data portal (http://xena.ucsc.edu/). Gene expression matrices for the GSE161533, GSE23400 (GPL97 platform), and GSE47404 cohorts were obtained from the Gene Expression Omnibus (GEO) database. Gene expression matrices and clinical data for cohorts GSE53624, GSE53625, and GSE19417 cohorts were downloaded. Autophagy genes (ATGs) were derived from the Autophagy Database (http://www.tanpaku.org/autophagy/) (**Supplementary Table 1**).

### Download of immunotherapy cohorts

The data for the GSE165252, GSE91061, and GSE176307 cohorts were downloaded from GEO. Visit http://researchpub.gene.com/IMvigor210CoreBiologies/ for more information on the IMvigor210 cohort (23). In addition, the data from the Gide2019 and Nathanson2017 cohorts were obtained from the TIDE (http://tide.dfci.harvard.edu/) database (24). The **Supplementary Table 2** presented these detailed data.

### Survival analysis

Clinical information (age, gender, stage, pathology, etc.) was collected for ESCA patients in the TCGA cohort. 185 samples were considered eligible after screening for transcriptomic and clinicopathological information. After excluding one duplicate sample and one sample with 0 days of follow-up, Kaplan-Meier analysis was performed on these 183 samples using the survival and survminer R packages. Univariate Cox regression analysis was conducted to screen out risk variables, of which p<0.2 were further included in multivariate Cox regression analysis.

### Construction of a nomogram

Variables with clinical significance and multivariate Cox regression p<0.05 were screened to establish a predictive nomogram. The regplot package was used to plot the nomogram. Calibration curves were calculated with the use of the rms package. Decision curve analysis (DCA) was conducted using the “stdca.R” function. Receiver operating characteristic (ROC) curve analysis was calculated using the timeROC R package (version 0.4) (25).

### Immunological characterization of the TME

We have obtained gene set labels including 28 types of immune cells to accurately evaluate the atlas of immune cells infiltrating in the TME. We calculated the enrichment fraction within each immune cell subtype for each individual using the single sample gene set enrichment analysis (ssGSEA) algorithm of the GSVA package. The anticancer immune response also is recognized as a stepwise multiplicity of processes called the cancer immunity cycles. By analyzing the 23 gene sets associated with the seven-step cancer immunity cycles, the researchers were able to explore the tumor immune phenotype (26). We received a total of 92 immune-related signatures from previous work (**Supplementary Table 3**) (10). The ssGSEA algorithm of the GSVA package was used to calculate the enrichment score (ES) of these immune-related signatures. We collected 23 chemokines and receptors from previous literature associated with the recruitment of myeloid-derived suppressor cells (MDSCs), tumor-associated macrophages (TAMs), and Treg cells (27). Some patients treated with immune checkpoint inhibitors (ICIs) may experience the side effect of cancer hyper-progression. We summarize several predictive genes for hyper-progression (28, 29). In addition, we have identified 22 inhibitory immune checkpoints that have therapeutic potential (30). The researchers used CIBERSORT, TIMER, QUANTISEQ, MCPCOUNTER, XCELL, EPIC, and other algorithms to quantify immune cell infiltration (TIMER 2.0 database, http://timer.cistrome.org/) (31).

### Predicting immunotherapy response

The TIDE algorithm, and the IPS score were used to investigate the value of CDKL3 in the prediction of response to immunotherapy. TIDE scores were calculated from the official TIDE website (http://tide.dfci.harvard.edu/). IPS scores were calculated using the IOBR package (version 0.99.9) (32).

### Construction and validation of the CrA risk score model

Spearman correlation analysis was used to filter the CDKL3-related ATGs (p-value<0.1). Further, Univariate Cox regression (p-value<0.2) and LASSO Cox regression were used to construct the appropriate signature. Using ‘lambda. min’ from the R package ‘glmnet’ to obtain the optimal lambda value. Finally, the model-derived CrA risk score could be calculated the following equation.


$$\text{CrA risk score}=\sum_{i=1}^{9}\;\beta i\;*\;Ei$$

βi is the risk factor and Ei is the expression of each gene. Kaplan-Meier analysis was used to examine the correlation between the CrA risk score and overall survival (OS).

### Tissue collection and Immunohistochemical staining (IHC)

Tissue samples were collected from 24 ESCC patients receiving neoadjuvant chemoradiotherapy (nCRT) plus surgery at Taizhou Hospital, Zhejiang Province, between November 2011 and December 2020. The samples from every patient included cancer tissues before neoadjuvant chemoradiotherapy and after surgery. Analysis of postoperative pathological tumors (pT) and postoperative pathological lymph nodes (pN) were based on pathological assessment after surgical treatment. The definition of postoperative pathological complete response (pCR) was negative postoperative pathological tumor and postoperative pathological lymph nodes (pT^-^N^-^), and postoperative pathological complete response (non-pCR) was defined as pT^+^ and/or pN^+^. The inclusion criteria were: (1) The pathologic diagnosis of the primary tumor was confirmed as ESCC; (2) Tissue samples were stored in the tissue bank of Taizhou Hospital; (3) Having a completed postoperative report of pathological assessment; (4) Having enough sample to perform immunohistochemical staining (IHC); (5) Patients who completed nCRT and surgery. The exclusion criteria were: (1) Non-ESCC patients; (2) Without a completed postoperative report of pathological assessment;(3) Without enough samples stored in the tissue bank of Taizhou Hospital. The flow of immunohistochemical staining (IHC) was carried out as we described in the previous study (22). Two observers blinded to the purpose of the study independently evaluated the stained sections. The score of CDKL3 expression was evaluated and calculated by independent blinded observers. The patients with scores > 8 were classified as a high CDKL3-expression group, otherwise as a low CDKL3-expression group.

### Cell cultures, macrophage induction

The human ESCC cell line KYSE-150 were obtained from the Shanghai Institute of Cell Biology, Chinese Academy of Sciences (Shanghai, China). Cells were maintained in RPMI 1640 with 10% FBS (Sigma, St Louis, USA) 100µg mL-1 streptomycin and 100µg mL-1 penicillin, 37°C, 5% CO2.THP-1 cells (ATCC, Manassas, USA), as human peripheral blood monocytes, were incubated with serum-free RPMI 1640 containing 200 nM PMA (Sigma, St Louis, USA) for 48h to induce M0 macrophage.

### Cells co-culture and macrophage polarization analysis

The supernatant harvested from 48h incubation of KD and NC groups of KYSE-150 was co-cultured with M0 macrophage in 24-well transwell plates (Millipore Co., Bedford, MA) for 72h. Macrophages without co-culture were set as the control. After polarization induction, macrophages were harvested and incubated with specific primary anti-bodies against relative surface markers (CD86 as an M1 marker, and CD206 as an M2 marker) on ice for 30 minutes. Then, these stained cells were resuspended in 400 μL of PBS after twice of cold PBS washing. Finally, flow cytometry (BD LSRII system, BD Biosciences, Franklin Lakes, USA) was applied to evaluate the specific surface markers. Simultaneously, macrophages were harvested to extract total RNA using Trizol reagent (Invitrogen, Carlsbad, CA) in accordance with instructions. qRT-PCR (Mx3000Ps, Biosystems Inc., Foster City, CA, USA) was performed after reverse transcription to cDNA (PrimeScript RT reagent Kit, Takara, Shiga, Japan). The PCR reaction condition was as follows: pre-denaturing at 95°C for 15s, then 45 cycles at 95°C for 5s, ended after 60°C for 30s. The internal reference used in this study was GAPDH. The primers of targeted genes were shown as follows: CDKL3: 5′- AAAGTGGGCAATTTGTCACCT-3′(forward), 5′-TTGGGGTGTTGAACTTGAGGA-3′(reverse); GAPDH: 5′-AGAAGGCTGGGGCTCATTTG-3′(forward), 5′-AGGGGCCATCCACAGTCTTC-3′ (reverse); IL-12: 5′-CCTTGCACTTCTGAAGA GATTGA-3′(forward), 5′-ACAGGGCCATCATAAAAGAGGT-3′(reverse); TNF-α: 5′-CCTCTCTC TAATCAGCCCTCTG-3′(forward), 5′-GAGGACCTGG GAGTAGATGAG-3′(reverse); IL-10: 5′-GACTTTAAGGGTTACCTGGGTTG-3′(forward), 5′-TCACATGCGCCTTGATGTCTG-3′(reverse); TGF-β: 5′-GGCCAGATCCTGTCCAAGC-3′(forward), 5′- GTGGGTTTCCACCATTAGCAC-3′(reverse). Relative gene expression was determined using the 2^-ΔΔCT^ method. In other ways, co-culture supernatants were collected and tested for specific cytokines (TNF-α and IL-12 as M1 markers, TGF-β and IL-10 as M2 marker) using ELISA kits (eBioscience, San Diego, USA) according to the manufacturer’s protocols.

### Confocal imaging of autophagosomes and autolysosomes

Cells were plated in 6-well chambers at 10000 cells/well followed by transfection with tandem fluorescently tagged LC3B (pLVX-Puro-RFP-GFP-hLC3B) lasting 24h. Then, indicated drugs were added to co-incubate with the cells for another 24 h. Subsequently, DAPI or Hochest was used to stain the cell nuclei for 15 min after the fixation with 4% Paraformaldehyde (PFA). Finally, autophagic flux was monitored and scanned by Pannoramic Midi (3DHistech, Budapeste, Hungary).

### Gene silencing

For transfection, cells were cultured in 6-well plates containing 5x10^5^ cells. Transient transfection of small interfering RNA (siRNA) was carried out using Lipofectamine 3000 Transfection Reagent (L3000015, Thermo Fisher Scientific, California, USA). siRNAs targeting human CDKL3 were obtained from GenePharma (Shanghai, China) and transfected into cells using Lipofectamine 3000 (Invitrogen, California, USA). The detailed sequences of siRNA were as follows: CDKL3 siRNA1 (KD), 5′-UCAGGAAAGAUGAAAGAAATT-3′, 5′-UUUCUUUCAUCUUUCCUGATT-3′; CDKL3 siRNA2 (KD), 5′-GCUGCAAAUCUCAGUUCAAAU-3′, 5′-UUGAACUGAGAUUUGCAGCCA-3′; CDKL3 siRNA3 (KD), 5′-AGUUCUUCCUCAAGUUCAACA-3′, 5′-UUGAACUUGAGGAAGAACUAC-3′; CDKL3 siRNA4 (KD), 5′-GACUAUCUUCACAGUAAUAAU-3′, 5′-UAUUACUGUGAAGAUAG UCAA-3′; NC siRNA (NC), 5′-UUCUCCGAACGUGUCACGUTT-3′, 5′-ACGUGACACGUUCGGA GAATT-3′.

### Western blot assay

Briefly, total proteins were extracted from ESCC cells and protein quantification was performed using the BCA protein assay kit (Beyotime, Shanghai, China). 10% SDS-PAGE was used to separate protein samples, transferred to PVDF membranes, and then the PVDF membranes were blocked with 5% skim milk for 1 hour. The membranes were then incubated overnight with mouse anti-CDKL3 antibody (Sigma-Aldrich, St. Louis, MO, USA) or rabbit anti-LC3B antibody (Cell Signaling Technology, Danvers, MA, USA) at 1:1000 dilution. Mouse anti-GAPDH antibody (Santacruz, Santa Cruz, CA, United States) (1:2000) was incubated overnight at 4°C as a control for the top sample. Finally, an HRP-coupled IgG antibody (Santacruz, Santa Cruz, CA, United States) was used as a secondary antibody. Signal bands were also quantified using ImageJ software.

### Ingenuity pathway analysis (IPA)

Our previous study analyzed the profile of differential expressed genes between KYSE-150-NC and KYSE-150-CDKL3-KD cells using GeneChip^®^ PrimeViewTM human gene expression arrays (22). In this study, we used IPA to perform gene enrichment analysis based on the results of CDKL3 expression-related differential gene expression.

### Statistical analysis

All statistical data analysis and graphs were performed using R software (version 4.2.1) and GraphPad Prism (version 8.0.2) for analysis. All experiments were replicated at least three times. Differences between the two groups were analyzed using a t-test or Wilcoxon test. Correlations between variables were examined using Spearman’s coefficient. Survival curves were plotted using the Kaplan-Meier method, and the log-rank test was used to compare between groups. Two-tailed statistical tests were applied and p < 0.05 was used to define as statistically significant (“NS” indicates no significant difference, *p<0.05, **p<0.01, ***p<0.001, and ****p<0.0001).

## Results

### CDKL3 expression pattern and mutation analysis

Expression data from the TCGA and GEO databases were comprehensively analyzed. In the TCGA database, we found no differences in CDKL3 expression between tumor and normal tissue of ESCA. Compared to the paired normal tissue samples in GEO databases, CDKL3 was highly overexpressed in the tumor tissues of ESCA (GSE161533 and GSE23400, (**Supplementary Figure 1A**). We obtained ESCA mutation data and divided patients into two groups based on CDKL3 expression. Patients with higher CDKL3 expression typically had a higher TP53 mutation rate (87% vs. 82%) and lower TTN (38% vs. 42%) and MUC16 (18% vs. 26%) mutation rates (**Supplementary Figures 1B, C**) than that of patients with lower CDKL3 expression.

### Elevated CDKL3 expression predicts poor clinical outcomes in ESCA patients

**Figure 2:**
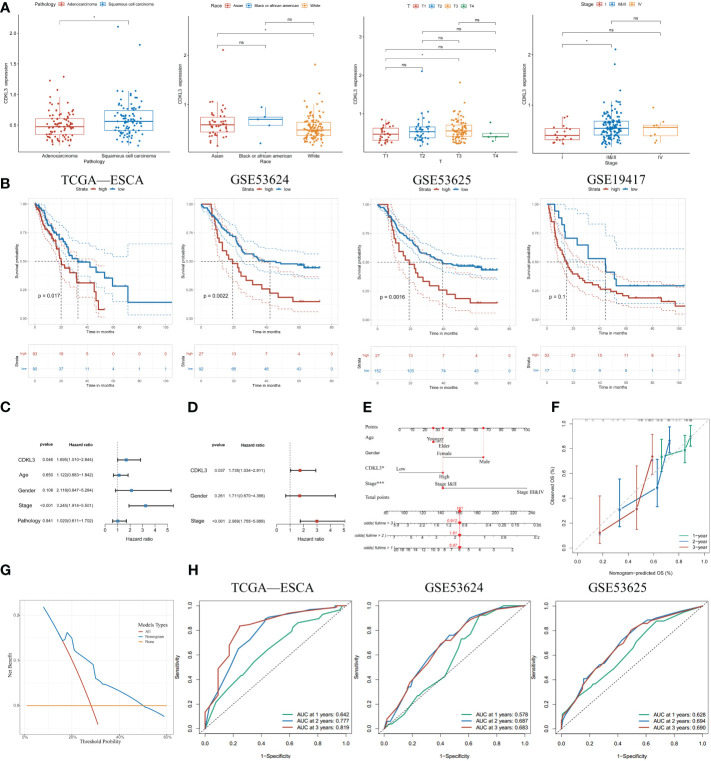
Increased expression of CDKL3 indicates poor prognosis. **(A)** The association between CDKL3 and pathology, race, T, and stage. **(B)** Prognosis of high and low CDKL3 expression groups in the TCGA, GSE53624, GSE53625, and GSE19417 cohorts. **(C)** Univariate analysis and **(D)** Multivariate analysis of CDKL3 expression and clinicopathological features in the TCGA cohort. **(E)** Nomogram for predicting the prognosis of ESCA patients. **(F)** Calibration plots indicate the predicted overall survival at 1, 2, and 3 years. **(G)** Decision curve analysis (DCA) of the nomogram. **(H)** Receiver operator characteristic (ROC) analysis of the nomogram in the TCGA, GSE53624, and GSE53625 cohorts. *p<0.05; ns, no significance.

To further determine the clinical significance of CDKL3 in ESCA patients, our study of clinical data from the TCGA-ESCA dataset revealed a significant association between high CDKL3 expression and pathology (squamous cell carcinoma vs. adenocarcinoma), race (Asian vs. White), T (T3 vs. T1) and tumor stage (stage II&III vs. stage I) (**Figure 2A**). This suggests that CDKL3 expression levels increase with increasing ESCA malignancy. To further understand the significance of CDKL3 in ESCA, we investigated the relationship of its expression with prognosis in ESCA patients. According to the median value of CDKL3 expression, patients were grouped into high- and low-expression cohorts. Log-rank test analysis then indicated that those with high CDKL3 expression had a worse outcome than patients with low CDKL3 expression in the TCGA-ESCA (n=183), GSE53624 (n=119), and GSE53625 (n=179) cohorts, while a similar but non-significant trend was found in the GSE19417 cohort (n=70) (**Figure 2B**). Univariate analysis showed that patients with high CDKL3 expression (HR:1.695, 95% confidence interval (CI):1.010-2.844, p=0.046) had shorter overall survival (**Figure 2C**). After adjustment for other confounders (gender and stage), multivariate analysis indicated that CDKL3 remained an independent prognostic risk factor in ESCA patients (HR:1.735, 95% CI:1.034-2.911, p=0.037) (**Figure 2D**). A nomogram was created to estimate the outcome of ESCA patients and the variables considered in the nomogram were age, gender, CDKL3, and stage (**Figure 2E**). The C-index value of the nomogram was 0.675 (95% CI: 0.635-0.715). The calibration curve showed the accuracy of the nomogram in the prediction of survival at 1, 2, and 3 years (**Figure 2F**). A decision curve analysis (DCA) was performed (**Figure 2G**), which suggested a good probability of diagnosis between a probability threshold of 20% and 50%. In conclusion, ROC analysis was performed to evaluate the sensitivity and specificity of this nomogram in the prediction of OS at 1, 2, and 3 years. The AUC for 1-, 2-, and 3-year OS were 0.642, 0.777, and 0.819 in the TCGA cohort, respectively. The AUC for 1-, 2-, and 3-year OS were 0.578, 0.687, and 0.683 in the GSE53624 cohort, respectively. The AUC for 1-, 2-, and 3-year OS were 0.628, 0.694, and 0.690 in the GSE53625 cohort, respectively (**Figure 2H**). These findings suggest that the expression level of CDKL3 can be considered a powerful prognostic predictor in ESCA patients.

### Correlation between CDKL3 and immune phenotype

**Figure 3:**
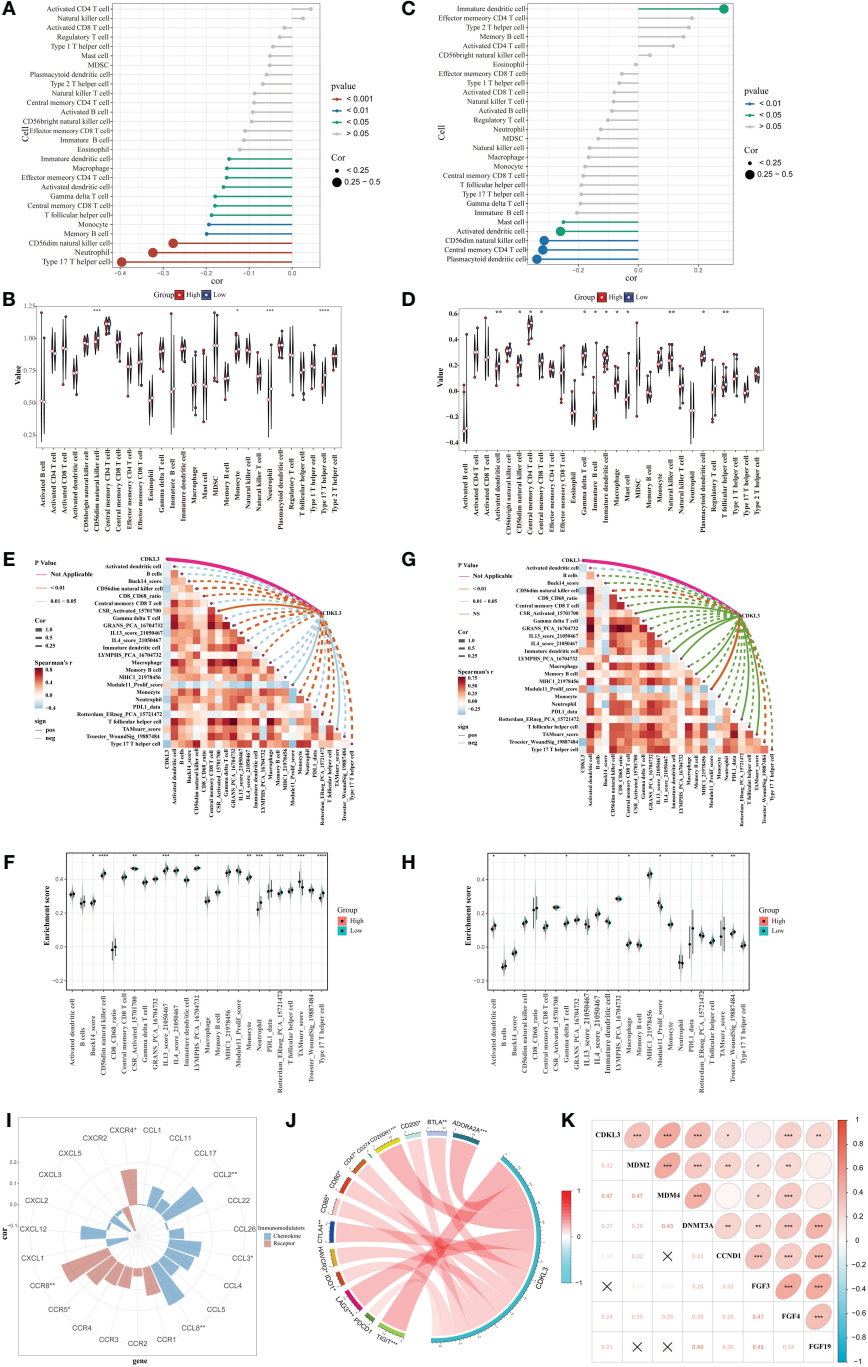
Correlation between CDKL3 and immunological characteristics in the TME. **(A)** Correlation of CDKL3 with infiltration levels of TIICs in the TCGA cohort. **(B)** Violin plots of infiltrating degrees of TIICs in the TCGA cohort. **(C)** Correlation of CDKL3 with infiltration levels of TIICs in the GSE47404 cohort. **(D)** Violin plots of infiltration levels of TIICs in the GSE47404 cohort. **(E)** Correlation of CDKL3 with 25 immune-related signatures in the TCGA cohort. **(F)** Violin plots of enrichment scores of immune-related signatures in the TCGA cohort. **(G)** Correlation of CDKL3 with 25 immune-related signatures in the GSE47404 cohort. **(H)** Violin plots of enrichment scores of immune-related signatures in the GSE47404 cohort. **(I)** Correlation of CDKL3 with chemokines and chemokine receptors. **(J)** Correlation of CDKL3 with inhibitory immune checkpoints. **(K)** Correlation of CDKL3 with hyperprogressive genes for immunotherapy. *p<0.05; **p<0.01; ***p<0.001; ****p<0.0001.

Spearman analysis indicated that CDKL3 expression was significantly and negatively related to the majority of tumor-infiltrating immune cells (TIICs) (**Figure 3A**). Patients were stratified into high and low CDKL3 expression groups according to median CDKL3 expression. The relative abundance of immunoreactive cells was markedly decreased lower in CDKL3 high-expressed group (**Figure 3B**). Using the GSE47404 cohort as a validation set, the results were highly consistent with the above findings (**Figures 3C, D**). In ESCA, CDKL3 expression was closely associated with the remodeling of the TME. In the TCGA cohort, CDKL3 expression was negatively correlated with the activity of step 4 of the cancer immunity cycles, i.e. immune cell trafficking to the tumor (**Supplementary Figure 2A**), which was further validated in the GSE47404 cohort (**Supplementary Figure 2B**). This explains why higher CDKL3 expression was associated with lower infiltration of immunoreactive cells.

ES heatmaps were presented for the correlation among CDKL3 and 92 immune-related signatures in the TCGA and GSE47404 cohorts (**Supplementary Figures 2C, D**). CDKL3 was strongly related to 25 immune-related signatures in the TCGA cohort, most of which were anti-tumor signatures that were negatively associated with CDKL3. Notably, CDKL3 had a significantly positive correlation with the ES of the TAM-related signature (TAMsurr_score). Furthermore, the ES for the anti-tumor signatures was considerably lower in the CDKL3 high expression group with a higher ES for the TAMsurr_score (**Figures 3E, F**). This finding was further validated in the GSE47404 cohort (**Figures 3G, H**). In summary, raised CDKL3 expression promoted the tumor immune phenotype to become a ‘cold’ type. Subsequently, the analysis about the relation between CDKL3 expression and suppressive TME-related chemokines and receptors (**Supplementary Table 4**) revealed that high CDKL3 expression positively correlated to the expression of chemokines (CXCL2, CXCL3, CCL8) and chemokine receptors (CXCR4, CCR5, CCR8) (**Figure 3I**).

### CDKL3 expression levels predict response to immunotherapy

**Figure 4:**
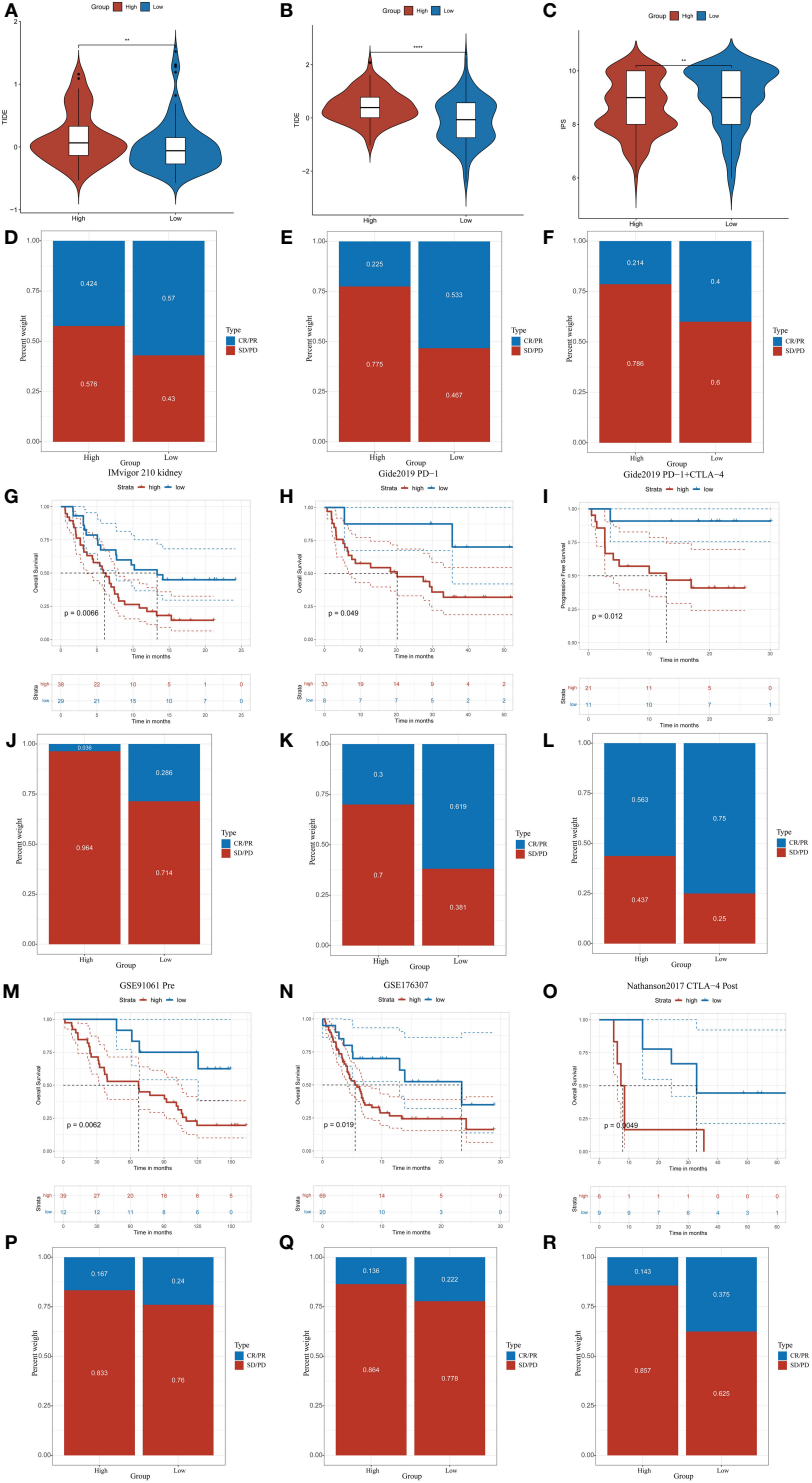
CDKL3 predicts response to immunotherapy. **(A, B)** TIDE scores in the TCGA and GSE53625 cohorts. **(C)** IPS scores in the TCGA cohort. **(D, E)** TIDE predicted immunotherapy response rates in the TCGA and GSE53625 cohorts. **(F)** Immunotherapy response rates based on GSE165252 (an immunotherapy cohort for ESCA). **(G–I, M–O)** Survival analysis of CDKL3 in the pan-cancer immunotherapy cohorts. **(J–L, P–R)** The proportion of pan-cancer immunotherapy responders in the high and low CDKL3 groups. **p<0.01; ****p<0.0001.

We explored the correlation of CDKL3 expression with that of 14 suppressive immune checkpoint inhibitors to determine the potential efficacy of CDKL3 in the prediction of response to ICIs in ESCA patients (**Figure 3J**). The results indicated that CDKL3 had a positive correlation with most of the inhibitory immune checkpoint inhibitors. Therefore, we suggest that CDKL3 may be a candidate biomarker for immunotherapy response prediction. It was discovered that the expression of CDKL3 exhibited a significant positive correlation to most of the hyper-progressive genes (**Figure 3K**), and CDKL3 may be associated with hyper-progression in immunotherapy. We also assessed the significance of CDKL3 as a predictor of immunotherapy response in ESCA patients using TIDE and IPS scores. Patients with low CDKL3 expression had significantly decreased TIDE scores and increased IPS scores, indicating that low CDKL3 patients have a reduced potentiality for immune escape and may have better efficacy with immune checkpoint inhibition therapy (**Figures 4A–C**). TIDE prediction showed that patients with lower CDKL3 expression group response to immunotherapy more significantly in the TCGA cohort (low group: 57.0%, 53/93 vs. high group: 42.4%, 39/92) (**Figure 4D**). Similarly, TIDE predicted that the low CDKL3 group in the GSE53625 cohort was more likely to respond to immunotherapy (low group: 53.3%, 48/90 vs. high group: 22.5%, 20/89) (**Figure 4E**). Based on GSE165252 (an immunotherapy cohort for ESCA), the group with CDKL3 low expression showed more superior response to the immunotherapy (low group: 40%, 6/15 vs. high group: 21.4%, 3/14) (**Figure 4F**). Thus, CDKL3 gene expression levels may help predict response to immunotherapy in ESCA patients. As there are fewer immunotherapy cohorts for ESCA, we investigated the role of CDKL3 in predicting the response to immunotherapy in other cancers. We discovered that the prognosis, including OS and progression-free survival (PFS), was worse for the high CDKL3 group in the metastatic urothelial cancer and melanoma cohorts (p < 0.05) (**Figures 4G–I, M–O**). Moreover, a trend toward a worse PFS was found in the high CDKL3 group of GSE176307 and Gide2019 PD-1 cohorts, while a trend toward a worse OS in the high CDKL3 group of Gide2019 PD-1+CTLA4 cohorts, yet the p-value was not statistically different (**Supplementary Figures 3A–C**). We also found that immunotherapy response rates were considerably lower among the high CDKL3 group than among the low CDKL3 group (**Figures 4J–L, P–R**). These results confirm that CDKL3 expression is a powerful indicator in pan-cancer immunotherapy cohorts.

### Establishment and validation of CrA-risk score model

**Figure 5:**
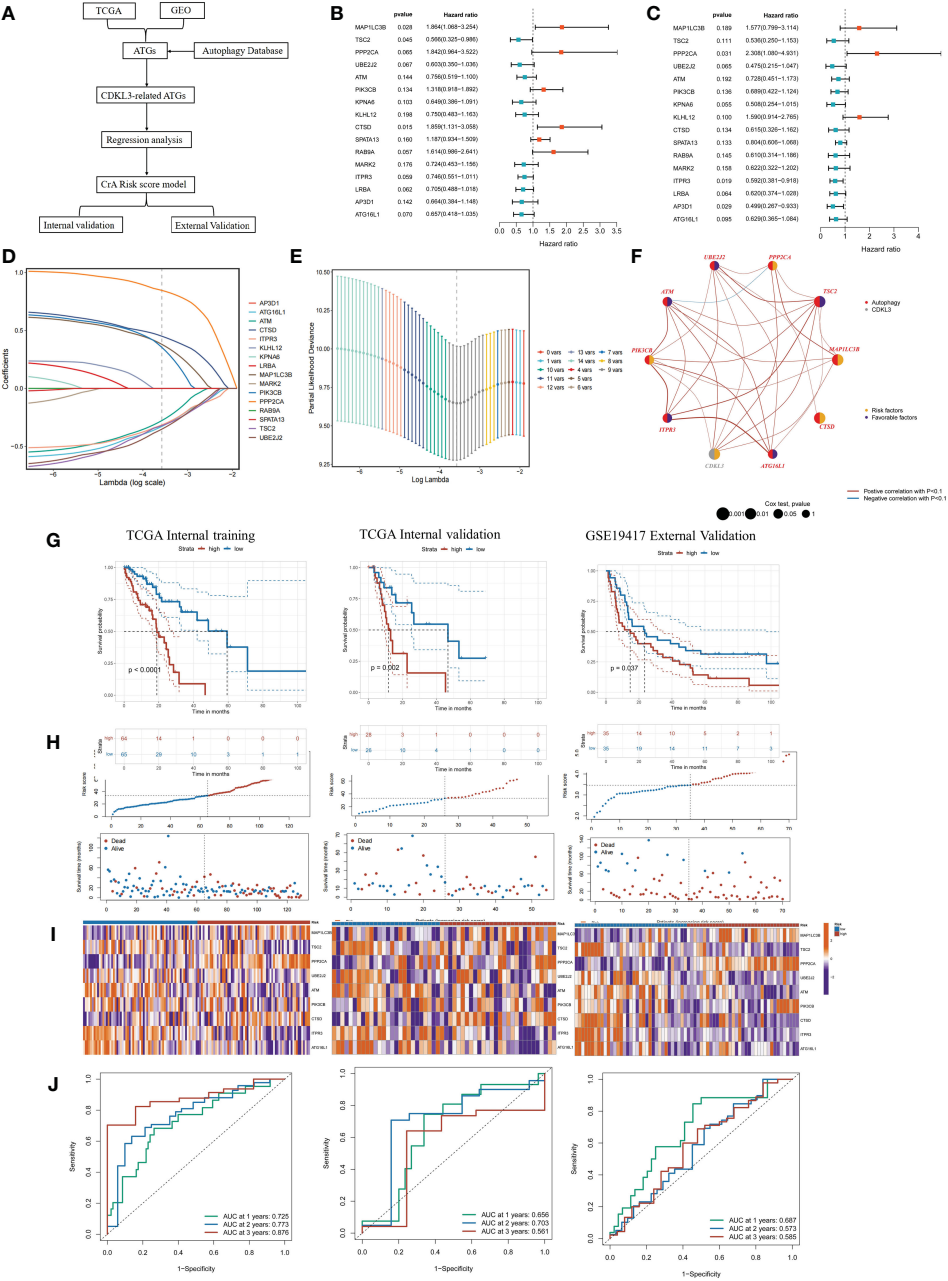
Construction and validation of the CrA risk score model. **(A)** Flowchart of the CrA risk score model. **(B)** Univariate analysis of 16 ATGs genes in the TCGA cohort. **(C)** Univariate analysis of 16 ATGs genes in the GSE19417 cohort. **(D)** Choosing the 9 model genes by LASSO Cox regression. **(E)** Cross-validation of the constructed signature. **(F)** Correlation and prognostic value of CDKL3 and model genes in TCGA. **(G)** Kaplan-Meier curves in TCGA internal training, TCGA internal validation and GSE19417 external validation cohorts. **(H)** Distribution of the CrA risk score adjusted for survival status and time in the TCGA internal training, TCGA internal validation and GSE19417 external validation cohorts. **(I)** Model gene expression heatmap from TCGA internal training, TCGA internal validation and GSE19417 external validation cohorts. **(J)** Receiver operator characteristic (ROC) analysis of the CrA risk score in the TCGA internal training, TCGA internal validation and GSE19417 external validation cohorts.

Utilizing 1183 ATGs downloaded from the autophagy database, we constructed and validated a CrA risk score model (**Figure 5A**). Correlation analysis and univariate COX regression analysis identified 16 genes at the output intersection of the TCGA and GSE19417 cohorts (MAP1LC3B, TSC2, PPP2CA, UBE2J2, ATM, PIK3CB, KPNA6, KLHL12, CTSD, SPATA13, RAB9A. MARK2, ITPR3, LRBA, AP3D1, ATG16L1). Univariate Cox regression revealed 16 predictive ATGs in the TCGA and GSE19417 cohorts (**Figures 5B, C**). The ESCA patients from the TCGA cohort were grouped into a training set (n = 129) and a validation set (n = 54) according to 7:3. Subsequently, a CrA risk score model for ESCA patients was developed via LASSO cox regression. Finally, 9 of the 16 ATGs were considered to be the best candidate genes (**Figures 5D, E**). The model of the CrA risk score was shown as the following.

CrA risk score = (0.380889116*MAP1LC3B exp) + (-0.321484102*TSC2 exp) + (0.846337455*PPP2CA exp) + (-0.351584102*UBE2J2 exp) + (-0.277601266*ATM exp) + (0.354855247*PIK3CB exp) + (0.450974523*CTSD exp) + (-0.315185584*ITPR3 exp) + (-0.327763971*ATG16L1 exp).

Genes involved in the signature included MAP1LC3B, TSC2, PPP2CA, UBE2J2, ATM, PIK3CB, CTSD, ITPR3, ATG16L1. The distribution of the above genes and CDKL3 on their respective chromosomes in ESCA was depicted in **Supplementary Figure 4A**. CNV alterations were prevalent in these genes. ATG16L1 showed the highest loss frequency, whereas PIK3CB showed the highest gain frequency (**Supplementary Figure 4B**). The correlation and prognostic impact of these genes in TCGA-ESCA were investigated (**Figure 5F**). Patients in the TCGA-ESCA internal training set (n=129), the TCGA-ESCA internal validation set (n=54) and the GSE19417 external validation set (n=70) were separated into high- and low-risk groups according to the median value of CrA-risk score model. Those belonging to the high-risk group of both the training and validation cohorts experienced shorter OS than those in the low-risk group (**Figures 5G–I**). In the TCGA-ESCA internal training set, ROC curves indicated that AUC values for 1-year, 2-year, and 3-year time points were 0.725, 0.773, and 0.876, respectively. The TCGA-ESCA internal validation and the GSE19417 external validation set also confirmed that the CrA risk score was highly reliable in predicting ESCA patients (**Figure 5J**). According to the Sankey plots, patients belonging to the high CDKL3 group were associated with the high-risk group and showed a tendency to have a poorer prognosis (**Supplementary Figure 4C**).

### Relationship between CrA risk score and immune infiltrating cells

**Figure 6:**
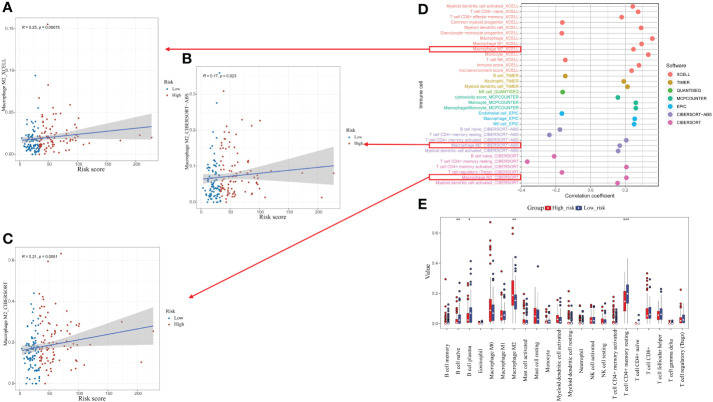
Relationship between the CrA risk score and tumor-infiltrating immune cells (TIICs). **(A–C)** Correlation between the CrA risk score and M2 macrophages based on XCELL, CIBERSORT-ABS and CIBERSORT algorithms. **(D)** Correlation between the CrA risk score and infiltrated TIICs. **(E)** The differences in TIICs levels between high- and low-risk groups. *p<0.05; **p<0.01; ***p<0.001.

The association between the CrA risk score and the level of TIICs was explored to further investigate the relevance of autophagy to the immune system in ESCA. Notably, the CrA risk score of ESCA patients had a positive correlation with M2 macrophage infiltration in three algorithms (**Figures 6A–C**). Moreover, there was a positive association between the CrA risk score and the level of multiple infiltrating immunosuppressive cells, which promote tumor progression, while negative with levels of anti-tumor immune cells (**Figure 6D**). The CIBERSORT algorithm showed that lower infiltration of immune-activating cells and higher infiltration of M2 macrophages were found in the high-risk group (**Figure 6E**). In other words, patients in the high-risk group had enhanced immunosuppression, which accounted for their worse prognosis.

### The relationship among CDKL3 expression, radiotherapy, and autophagy predicts in ESCC patients

**Figure 7:**
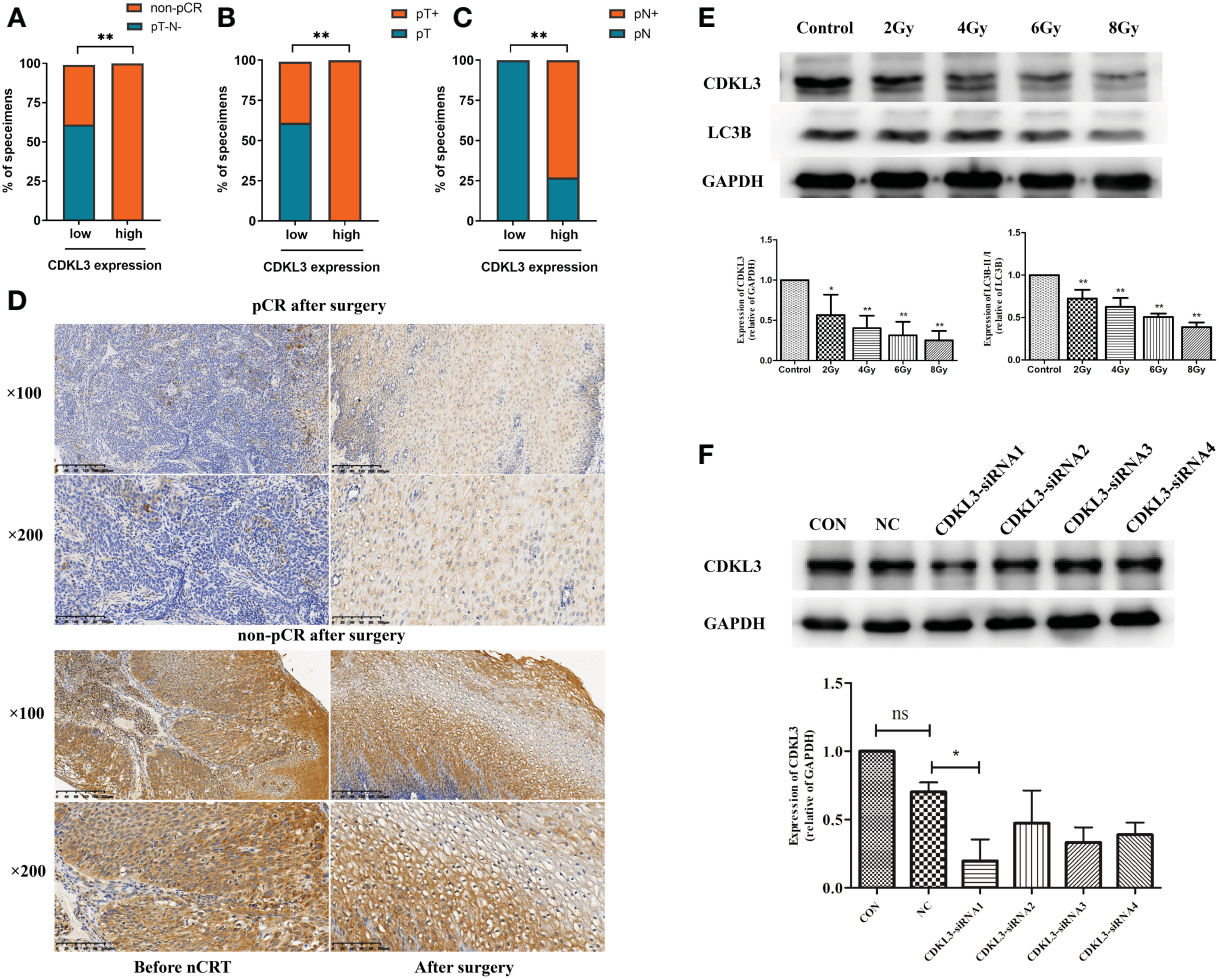
Relationship among CDKL3 expression, radiotherapy, and autophagy predicts in ESCC patients. **(A–C)** Correlation between high and low expression of CDKL3 and pathological complete response (pCR), pathological lymph node (pN), and pathological tumor (pT). **(D)** Representative immunohistochemical images of CDKL3 staining before and after nCRT in ESCC patients with pCR or non-pCR after surgery. The scale bars correspond to 200 μm (magnification ×100) and 100 μm (magnification ×200). **(E)** Expression levels of CDKL3 and LC3B in KYSE-150 cells after 0, 2, 4, 6, and 8 Gy radiotherapy were evaluated by western blot. **(F)** The expression of CDKL3 was detected in the CON group, NC group, CDKL3-siRNA1 group, CDKL3-siRNA2 group, CDKL3-siRNA3 group, and CDKL3-siRNA4 group by western blot. *p<0.05; **p<0.01; ns, no significance.

The results of the western blot demonstrated that radiotherapy caused a significant dose-dependent reduction in the expression levels of both CDKL3 and the autophagy marker LC3B (**Figure 7E**). To study the role of CDKL3 in autophagy and macrophage polarization, KYSE-150 cells were transfected with CDKL3-siRNAs. KYSE-150 transfected with CDKL3-siRNA1 showed distinctly reduced CDKL3 expression, and KYSE-150-CDKL3-siRNA1 (defined as KD group) was used for further study (**Figure 7F**). Analysis of 24 samples from ESCC patients receiving nCRT found that low pre-treatment CDKL3 expression was positively related to pCR (pT-N-) (**Figure 7A**), pT- (**Figure 7B**), and pN- (**Figure 7C**) (p<0.01). The typical staining of CDKL3 in ESCC patients with pCR or non-pCR was significantly different. That is, ESCC subjects with higher CDKL3 levels had a poorer response to nCRT than ESCC subjects with lower CDKL3 levels (**Figure 7D**).

### CDKL3 expression affects autophagy induction in ESCC

**Figure 8:**
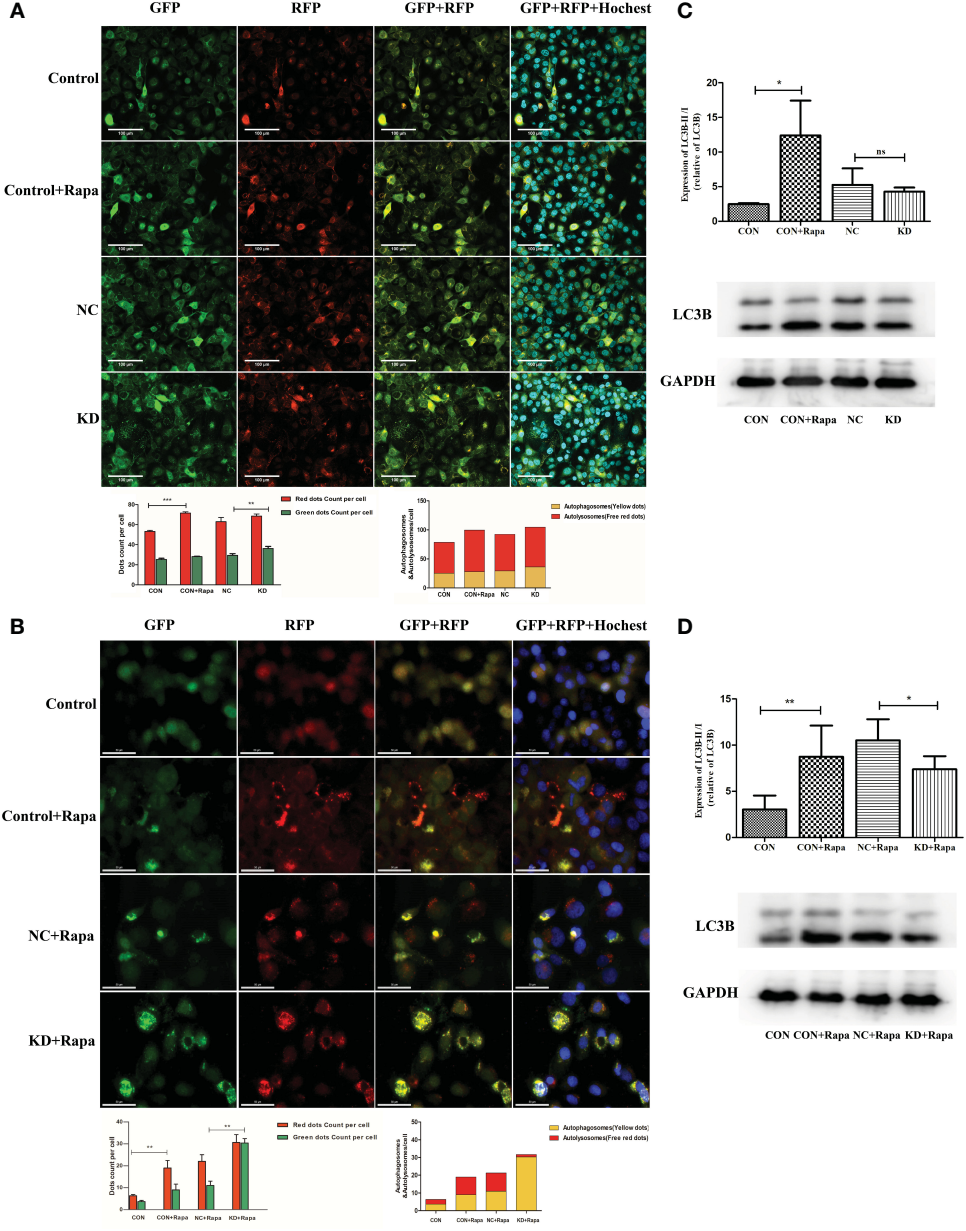
Effect of CDKL3 expression on autophagy in ESCC. **(A)** Expression of GFP, RFP, GFP+RFP, and GFP+RFP+Highest was detected by immunofluorescence. Representative co-staining images of the control group, Rapa group, NC group, and KD group. The scale bars correspond to 100 μm. **(B)** Expression of GFP, RFP, GFP+RFP, and GFP+RFP+DAPI was detected by immunofluorescence. Representative co-staining images of the control group, Rapa group, NC+Rapa group, and KD+Rapa group. The scale bars correspond to 50 μm. **(C)** The expression of LC3B in the CON group, CON+Rapa group, NC group, and KD group was assessed by western blot. **(D)** Expression of LC3B in the CON group, CON+Rapa group, NC+Rapa group, and KD+Rapa group was assessed by western blot. *p<0.05; **p<0.01; ***p<0.001; ns, no significance.

The addition of the autophagy inducer Rapa significantly increased autophagosomes, autophagic flow toward autophagic lysosomes and relative dots count red/green compared to CON (p<0.05). Relative dots count red/green decreased in KD vs. NC group (p<0.05), but the difference in values was within 20%, suggesting that CDKL3 knockdown alone may not have a significant effect on the flow of autophagosomes to autophagic lysosomes in KYSE150 cells (**Figure 8A**). The red/green count per cell was increased by the addition of the autophagy inducer Rapa in comparison to the CON group (p<0.05), indicating autophagic flow to autophagosomes. Compared to the NC+Rapa group, the KD+Rapa group had less red/green (p<0.05), significantly more autophagosomes (yellow dots) and significantly fewer autophagolysosomes (free red dots), suggesting that CDKL3 knockdown can significantly inhibit the flow of autophagosomes to autophagolysosomes in the autophagy-induced activated state of KYSE150 cells, i.e. inhibit autophagy induction (**Figure 8B**). The addition of the autophagy inducer Rapa significantly increased the autophagy marker LC3B compared to CON (p<0.05). Compared to the NC group, LC3B tended to decrease in the KD group, but was not statistically different. This suggests that CDKL3 knockdown alone may not have a significant effect on autophagy induction in KYSE150 cells (**Figure 8C**). LC3B was significantly higher in the CON+Rapa group than in the CON group. LC3B levels were also significantly lower in the KD+Rapa group than in the NC+Rapa group. It is suggested that CDKL3 knockdown can significantly inhibit autophagy induction in KYSE150 cells in the activated state of autophagy induction (**Figure 8D**). The above results suggested that downregulation of CDKL3 could inhibit autophagy activation.

### CDKL3 downregulation in ESCC promotes M1-type macrophage polarization

**Figure 9:**
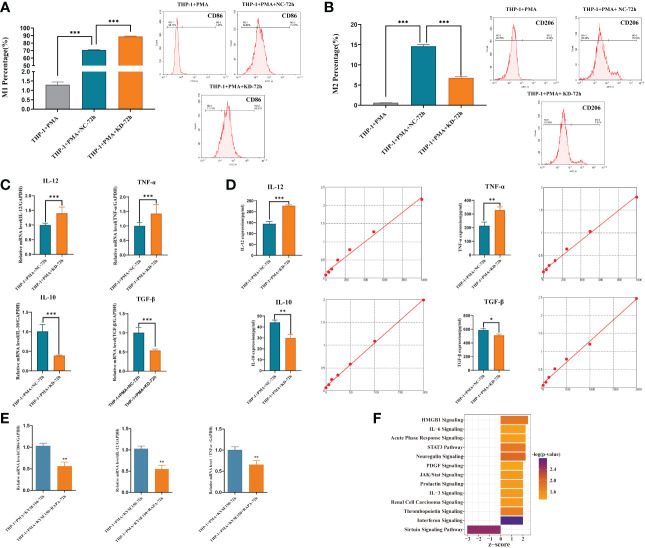
Effect of CDKL3 expression and autophagy activation on macrophage polarization. **(A)** The differences of M1 macrophages in the THP-1+PMA group, THP-1+PMA+NC-72h group, and THP-1+PMA+KD-72h group were evaluated by flow cytometry. **(B)** The differences of M2 macrophages in the THP-1+PMA group, THP-1+PMA+NC-72h group, and THP-1+PMA+KD-72h group were analyzed by flow cytometry. **(C, D)** Cytokine secretion (type M1: IL-12, TNF-α; type M2: IL-10, TGF-β) in THP-1+PMA+ NC-72h group and THP-1+PMA+KD-72h group were detected by qRT-PCR and ELISA, respectively. **(E)** Macrophage polarization markers (M1: CD86) and cytokine secretion (M1: IL-12, TNF-α) in THP-1+PMA+ KYSE150-72h group and THP-1+PMA+ KYSE150+RAPA-72h group were detected by qRT-PCR. **(F)** Downregulation of CDKL3 activates the Interferon (IFN) pathway, according to Ingenuity Pathway Analysis (IPA). The vertical coordinate is the pathway name and the horizontal coordinate is z-score (|z-score| ≥ 2 and -log(p-value) > 1.3). *p<0.05; **p<0.01; ***p<0.001.

THP-1 cells were induced into macrophages by PMA and then co-cultured with cultures of harvested KYSE-150-siCDKL3 cells for 72 h. Macrophage polarization (M1: CD86; M2: CD206) was detected by flow cytometry (**Figures 9A, B**) as well as qRT-PCR (**Figure 9C**), ELISA assay (**Figure 9D**) for cytokine secretion (M1: IL-12, TNF-α; M2: IL-10, TGF-β). The findings clearly indicated downregulation of CDKL3 expression in ESCC greatly promoted M1-type polarization and cytokine secretion in macrophages.

### Activation of autophagy in ESCC inhibits M1-type polarization of macrophage

KYSE-150 cells were induced with Rapa, an autophagy inducer, for 12 hours and then substituted with Rapa-free medium for another 12 hours. The cell supernatant was obtained and co-cultured with M0 macrophages for 72hours, and then RNA was extracted to detect macrophage polarization markers (M1: CD86) and cytokine secretion (M1 type: IL-12, TNF-α) by qRT-PCR. The results showed that ESCC cell supernatant after autophagy activation could inhibit macrophage M1-type polarization and cytokine secretion (**Figure 9E**).

### Downregulation of CDKL3 expression in ESCC activates the Interferon (IFN) pathway

IPA bioinformatics pathway analysis of KYSE-150-NC versus KYSE-150-CDKL3-KD cells revealed that the Interferon (IFN) pathway was significantly activated (**Figure 9F**). Therefore, we hypothesized that the high expression of CDKL3 in ESCC may attenuate anti-tumor immunity by inhibiting the IFN pathway.

## Discussion

We have previously published results showing that CDKL3 is highly overexpressed in ESCC and has a worse prognostic value (19, 22). Based on the public database of ESCA (mainly adenocarcinoma), the results of this study also showed that CDKL3 was highly expressed and associated with shorter survival. Recent studies suggested that upregulated CDKL3 expression is critical for promoting tumor development and poor prognosis in various solid tumors, including glioma and prostate cancer (20, 21). For example, Cui et al. found that overexpression of CDKL3 in glioma cells promotes cell proliferation and that RRM2 is a potential target of CDKL3. Upregulation of CDKL3 expression in glioma tissue independently predicts poor patient prognosis (20). Jiang et al. found that reducing CDKL3 levels substantially hindered cell proliferation and migration while promoting apoptosis and G2 cell cycle blockade in prostate cancer (21). Mutation analysis identified that those with high CDKL3 expression had more TP53 mutations and fewer TTN and MUC16 mutations. TP53 is linked to a poorer outcome in ESCA. Patients with TTN, MUC16 mutations have a higher tumor mutation load and may benefit from immunotherapy (33–35). As a result, patients with a high level of CDKL3 expression may benefit less from immunotherapy and result in a worse prognosis.

It is necessary to explore the reasons why CDKL3 represents a poor prognostic factor in ESCA. Building on previous research, our study provided the first look at the relationship between CDKL3 and the TME, autophagy, and response to immunotherapy in ESCA. We hypothesized that CDKL3 may alter tumor immunogenicity and immune infiltrating cells within the TME by influencing autophagy induction, thereby affecting immunotherapy patient response and prognosis in ESCA.

The TME comprises a multitude of distinct immune cell populations. TIICs may play a crucial effect on carcinogenesis and influenced tumor response to immunotherapy (36). The concept of the cancer immunity cycles was introduced by Chen and Mellman (37). The eventual killing of tumor cells by anti-tumor immune cells is the result of a series of seven steps accompanied by positive and negative regulation. Step 4 is the phase that activating T cells transfers into the circulation and migrates to the tumor, which is related to the infiltration level of TIICs (37). In this study, we observed that higher CDKL3 expression was related to reduced infiltration of a variety of immunoreactive cells. In the cancer immunity cycles, CDKL3 expression showed a negative correlation with the activity of step 4, and we speculated that CDKL3 may reduce the infiltration level of TIICs by inhibiting step 4. TAMs primarily promote the malignant transformation of tumors through the release of various factors. Recent studies have shown that TAM-derived CCL22 can activate the FAK signaling axis in tumor cells, thereby promoting ESCC progression (38). In our study, CDKL3 was significantly and positively associated with the TAM related signature (TAMsurr_score). Recruitment of different types of immune cell subpopulations in the TME is associated with chemokines and chemokine receptors. We collected chemokines and receptors associated with suppressive TME from previous study (27). These chemokines and receptors were associated with the recruitment of MDSCs (CXCL2, CXCL3), TAMs (CXCR4), Treg cells (CCR5), Treg cells (CCL8, CCR8). This suggested that CDKL3 may reshape the TME by regulating these chemokines and chemokine receptors, leading to the infiltration of immunosuppressive cells, ultimately affecting the response to immunotherapy and the promotion of tumor progression.

Research in tumor immunotherapy has progressed significantly in the last few years, and the application of ICIs has become a unique therapeutic approach for a variety of malignancies, including ESCA (4–7). It is crucial to find predictive markers for immunotherapy in ESCA. A recent meta-analysis included 5,257 patients with advanced ESCA who were treated with ICIs. The benefit of ICIs in the reduction of the risk of death in patients with ESCA was dependent on the PD-L1 CPS status. Further studies of immunotherapy biomarkers in the CPS <10 subgroup are needed (8). Our study also investigated CDKL3 as a candidate biomarker to predict response to immunotherapy. The findings indicated that CDKL3 showed a positive correlation with most of the inhibitory ICIs. However, no statistically significant correlation was found between CDKL3 and CD274 (PD-L1). The up-regulated inhibitory immune checkpoint of TME is associated with decreased anti-tumor immunity (30). This explains the poorer prognosis of ICIs in those with higher CDKL3 expression. The TIDE and IPS scores were used to assess how ESCA patients responded to immunotherapy. Poor response to immunotherapy in patients with high CDKL3 expression was also demonstrated in pan-cancer immunotherapy cohorts.

Both radical chemoradiotherapy and neoadjuvant chemoradiotherapy are the main anti-tumor treatment modalities for patients with locally advanced ESCA (39–41). There is emerging evidence that chemoradiotherapy may remodel the TME and thus interfere with the efficacy of immunotherapy (11, 12). Patients with ESCA who have a high rate of pCR after surgery have a favorable prognosis (39, 40). Considering that neoadjuvant immunotherapy combined with chemotherapy (or chemoradiotherapy) has only been used in a small number of clinical trials (42, 43). Therefore, we collected tumor samples from ESCA patients undergoing nCRT. The findings indicated that those expressing high levels of CDKL3 had a poorer response to chemoradiotherapy.

We have previously reported that CDKL3 has a regulatory relationship with ATG5, a gene that regulates autophagy, in KYSE-150 cells (22). Autophagy is essential for tumor migration, invasion, and tumor immunity, and it is regulated by chemoradiotherapy (14, 15). Immune cell subpopulations whose survival, activation, differentiation, and function in the TME are linked to the autophagy pathway (44). Recent reports indicated that inhibition of autophagy restores cell surface MHC-I levels, increases antigen presentation, and enhances the anti-tumor response. The anti-tumor effect of autophagy inhibition was dependent on CD8+ T cells and cell surface MHC-I expression. ICIs combined with autophagy inhibitors enhanced anti-tumor immune responses (45). Autophagy activation has also been associated with chemoradiotherapy resistance in ESCC, leading to poor patient prognosis. Xia et al. found that Nrf2 enhances radiation resistance through the targeting of CaMKIIα and subsequent activation of autophagy in ESCC (46). Our further studies also confirmed that radiotherapy affects autophagy activation, and the expression of CDKL3 affects autophagy induction. We developed a CrA risk score based on public databases and validated it in internal and external cohorts.

Macrophages are diverse and plastic and can polarize into different phenotypes and thus perform different functions in response to different stimuli. M1 macrophages have pro-inflammatory and anti-tumor activity. M2 macrophages may be involved in the immune escape of tumor cells due to their inhibition of inflammation and concomitant promotion of tumor proliferation (47). The researchers found that USP19 promoted autophagy and thus downregulated NLRP3 inflammasome activation. And USP19 promoted M2 macrophage polarization (48). Tumor cells could also induce M2 polarization by transferring genetic information via exosomal non-coding RNAs (49). Our study found that patients with a high CrA risk score had higher infiltration levels of M2 macrophages based on the XCELL, CIBERSORT-ABS, and CIBERSORT algorithms. In another study, the Necroptosis-Pyroptosis Genes (NPG) scores established for prognostic prediction were found to be negatively correlated with infiltrating M2 Macrophage in patients with clear cell renal cell carcinoma (ccRCC) by the CIBERSOR algorithm (50). Moreover, this study showed that CDKL3 knockdown in KYSE150 cells could significantly inhibit autophagy induction in an autophagy-induced activated state. ESCC cells with downregulated CDKL3 could secrete some soluble factors or proteins to promote M1 macrophage polarization. Activation of autophagy in ESCC inhibited macrophage M1 polarization. This suggests that high CDKL3 expression in ESCC cells may be associated with the activation of autophagy, which promotes macrophage M2 polarization. Lin et al. also found that silencing IL4I1 in ccRCC cell lines (786-O, 769-P) could inhibit M2-like macrophage polarization by indirectly co-culturing with M0 macrophages (51). These results suggest that tumor cells with specific altered genes might influence immune cell infiltration and functional polarization.

IFN-γ exerts its biological effects mainly through the JAK/STAT pathway by activating intracellular signaling networks (52). Grasso et al. found that this conserved IFN-γ transcriptome response enhanced the anti-tumor immune response in melanoma (53). Our study found that downregulation of CDKL3 expression in ESCC activated the IFN pathway. This provided a different perspective on the mechanism by which high CDKL3 expression leads to attenuated anti-tumor immunity.

The ESCA samples in TCGA were from a Western population. However, there are differences in the pathology of ESCA between Eastern and Western populations, in particular, squamous cell carcinoma is the main pathological subtype in Eastern patients while the vast majority of Western patients are adenocarcinoma (54). There are still some limitations and deficiencies in this study. The sample size of patients with ESCA retrieved from the TCGA and GEO databases was limited, especially for ESCC. Although bioinformatics analysis was conducted in ESCA patients including ESCC and esophageal adenocarcinoma, the vitro study evidence only confirmed the role of CDKL3 in ESCC cell lines while lacking data in esophageal adenocarcinoma. It is necessary to further distinguish the role of CDKL3 in ESCC patients from esophageal adenocarcinoma patients. The potential function of CDKL3 in the modulation of tumor microenvironment and autophagy has been initially identified in this study, but a further prospective exploration needs to be designed to confirm. Moreover, it is significant to investigate and compare the differences in the atlas of immune infiltrating cells, such as specific T cells and macrophages, of the ESCA population with different CDKL3 expression in the clinic. In summary, our next step is to collect clinical samples from ESCA patients receiving immunotherapy and conduct further research in multicenter cohorts in China.

## Conclusions

Overall, CDKL3 may play an important role in anti-tumor immunity by regulating autophagy to promote the formation of immunosuppressive TME, thus playing a critical role in the prognosis of ESCA.

## Data availability statement

The datasets presented in this study can be found in online repositories. The names of the repository/repositories and accession number(s) can be found in the article/**Supplementary Material**.

## Ethics statement

The studies involving humans were approved by the Institutional Review Board of Taizhou Hospital (K20230833). The studies were conducted in accordance with the local legislation and institutional requirements. The participants provided their written informed consent to participate in this study.

## Author contributions

YB: Data curation, Formal analysis, Investigation, Methodology, Software, Validation, Visualization, Writing – original draft, Writing – review & editing. JL: Data curation, Formal analysis, Investigation, Validation, Visualization, Writing – review & editing. SQ: Data curation, Software, Writing – review & editing. FJ: Data curation, Software, Writing – review & editing. CZ: Data curation, Investigation, Software, Writing – review & editing. HY: Conceptualization, Supervision, Validation, Visualization, Writing – review & editing. SZ: Conceptualization, Formal analysis, Funding acquisition, Supervision, Validation, Visualization, Writing – original draft, Writing – review & editing.

## Supplementary material

Supplementary Figure 1 Differential expression and mutational analysis of CDKL3. **(A)** CDKL3 expression differences between normal and tumor tissues in TCGA, GSE161533, and GSE23400 datasets. **(B, C)** Oncoplot of the top 20 most mutated genes between high and low CDKL3 groups.

Supplementary Figure 2 Association of CDKL3 with cancer immunity cycles and immune-related signatures. **(A, B)** Correlation between CDKL3 and cancer immunity cycles in TCGA and GSE47404 cohorts. **(C, D)** Heat maps of CDKL3 and 92 immune-related signatures in TCGA and GSE47404 cohorts.

Supplementary Figure 3 Survival analysis of CDKL3 in the pan-cancer immunotherapy cohorts. **(A)** GSE176307 **(B)** Gide2019PD-1 **(C)** Gide2019PD-1+CTLA-4.

Supplementary Figure 4 The landscape in CDKL3 and the model genes in ESCA. **(A)** Circos plot of chromosomal distribution of CDKL3 and model genes. **(B)** CNV frequency of CDKL3 and model genes. The horizontal axis represents the change in frequency. **(C)** Sankey diagram of the relationship between CDKL3 group, risk group, and survival status.
