# Supplementary figures and images for: CDKL3 shapes immunosuppressive tumor microenvironment and initiates autophagy in esophageal cancer
Source: Front Immunol. 2024 Mar 18;15:1295011. doi: 10.3389/fimmu.2024.1295011 (PMC10982402; doi:10.3389/fimmu.2024.1295011)

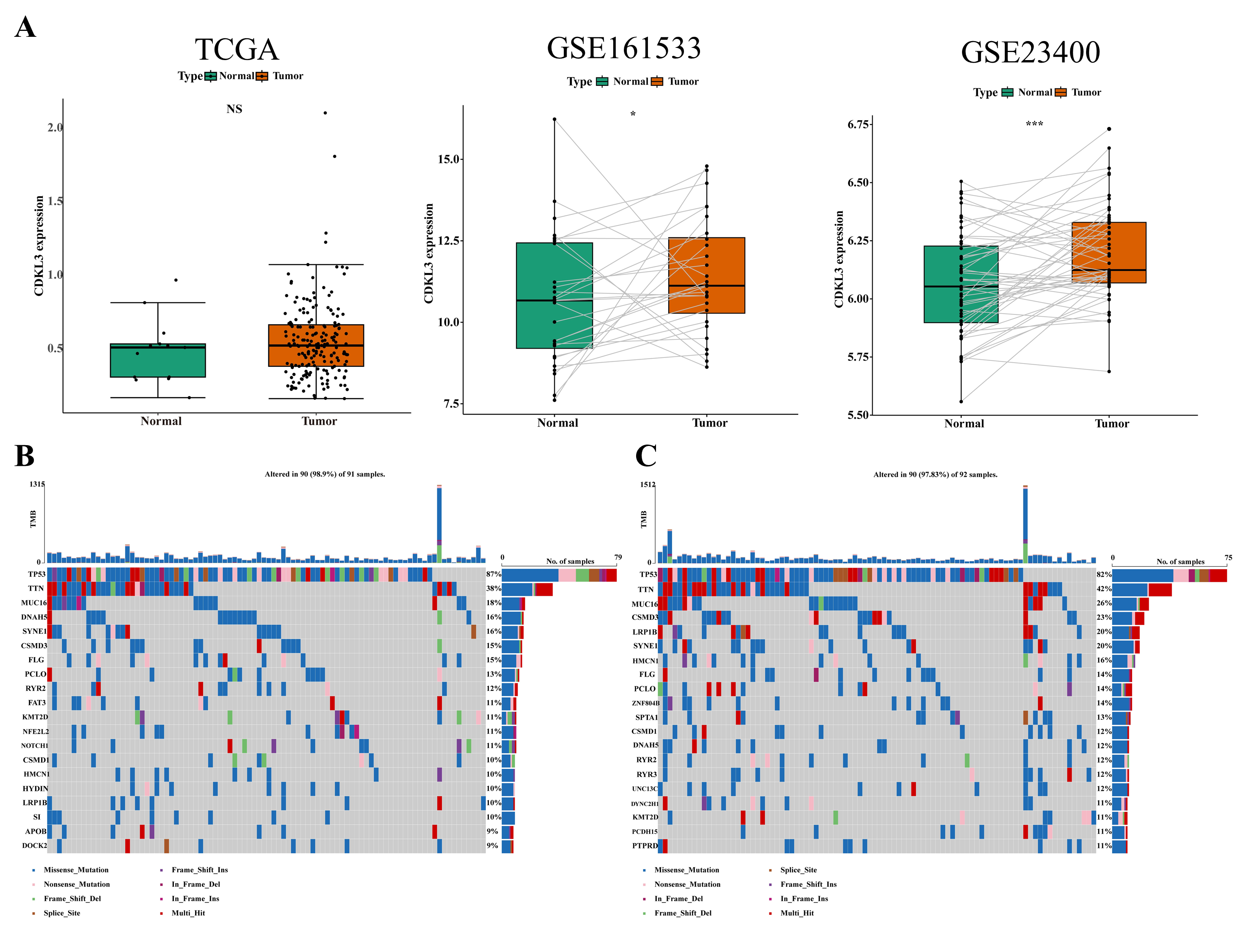

Supplement: Supplementary Figure 1 — Differential expression and mutational analysis of CDKL3. (A) CDKL3 expression differences between normal and tumor tissues in TCGA, GSE161533, and GSE23400 datasets. (B, C) Oncoplot of the top 20 most mutated genes between high and low CDKL3 groups. [file Image_1.tif]

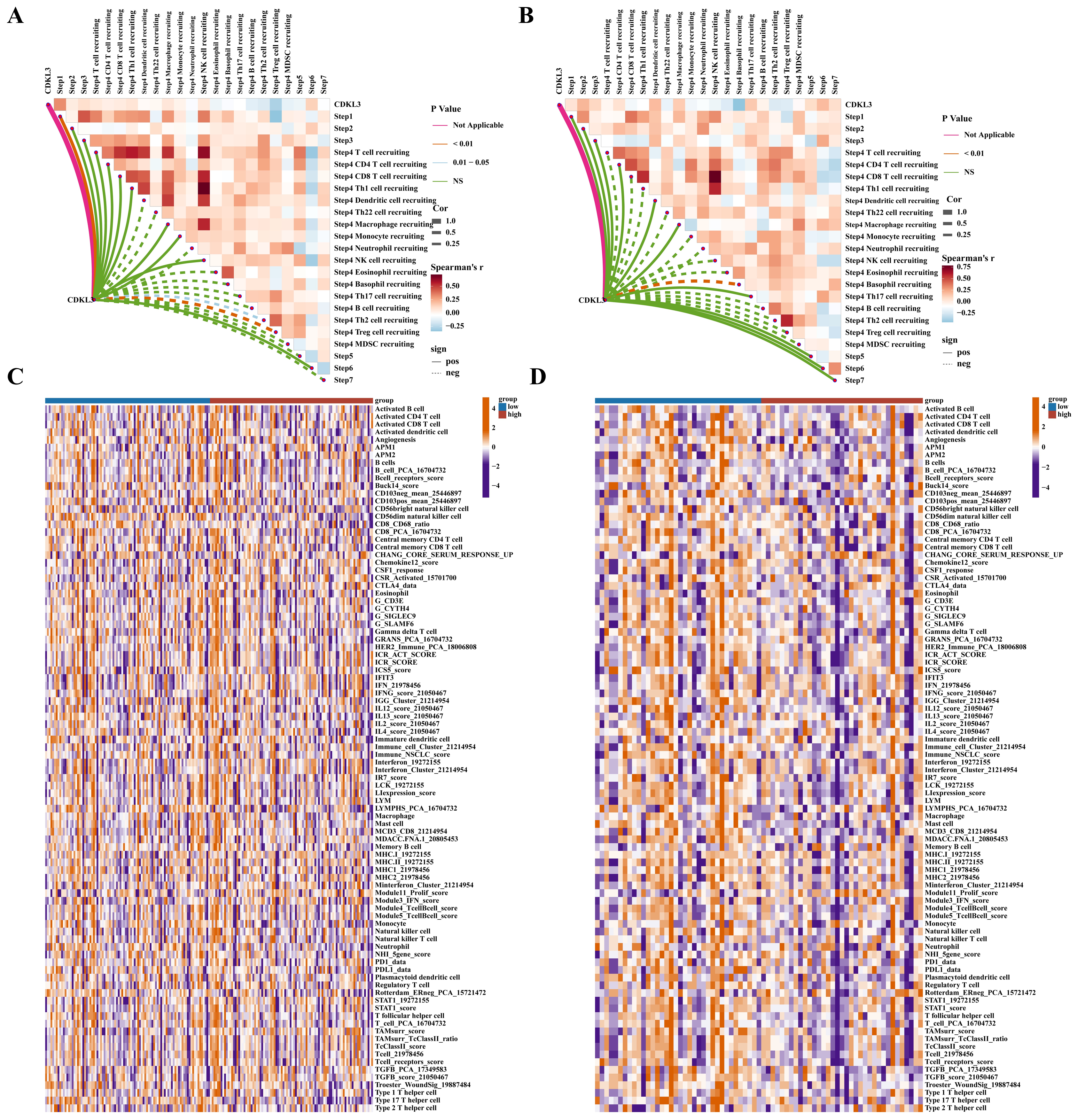

Supplement: Supplementary Figure 2 — Association of CDKL3 with cancer immunity cycles and immune-related signatures. (A, B) Correlation between CDKL3 and cancer immunity cycles in TCGA and GSE47404 cohorts. (C, D) Heat maps of CDKL3 and 92 immune-related signatures in TCGA and GSE47404 cohorts. [file Image_2.tif]

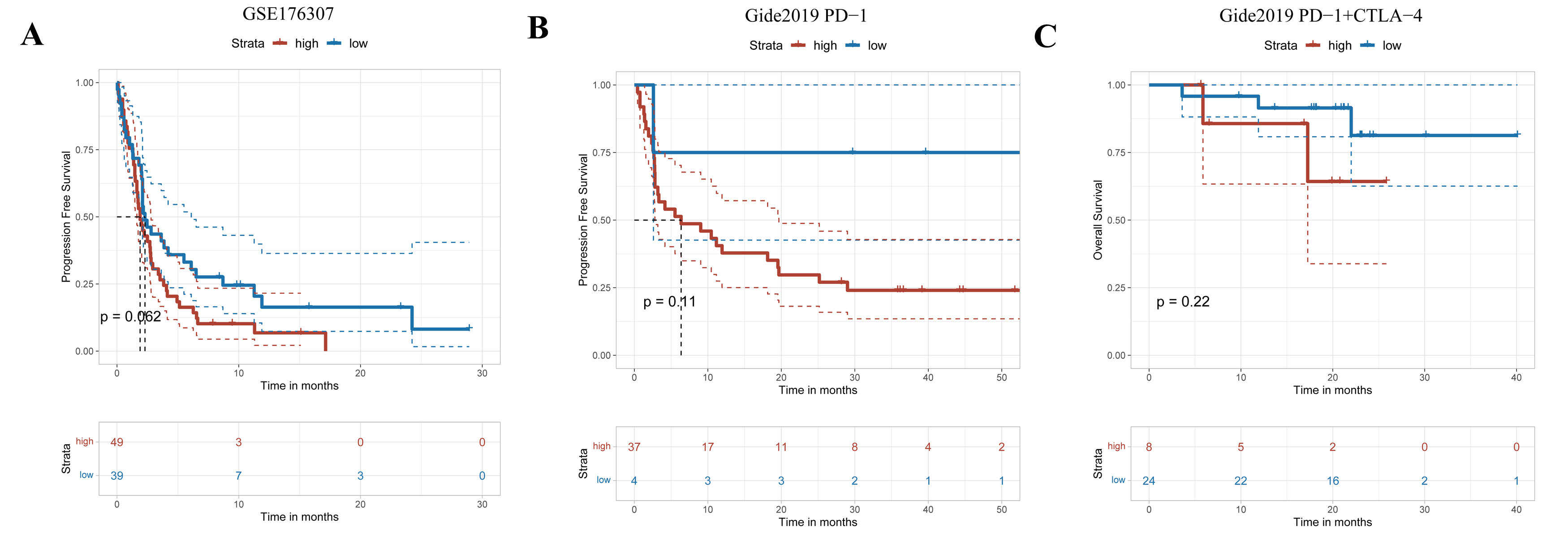

Supplement: Supplementary Figure 3 — Survival analysis of CDKL3 in the pan-cancer immunotherapy cohorts. (A) GSE176307 (B) Gide2019PD-1 (C) Gide2019PD-1+CTLA-4. [file Image_3.tif]

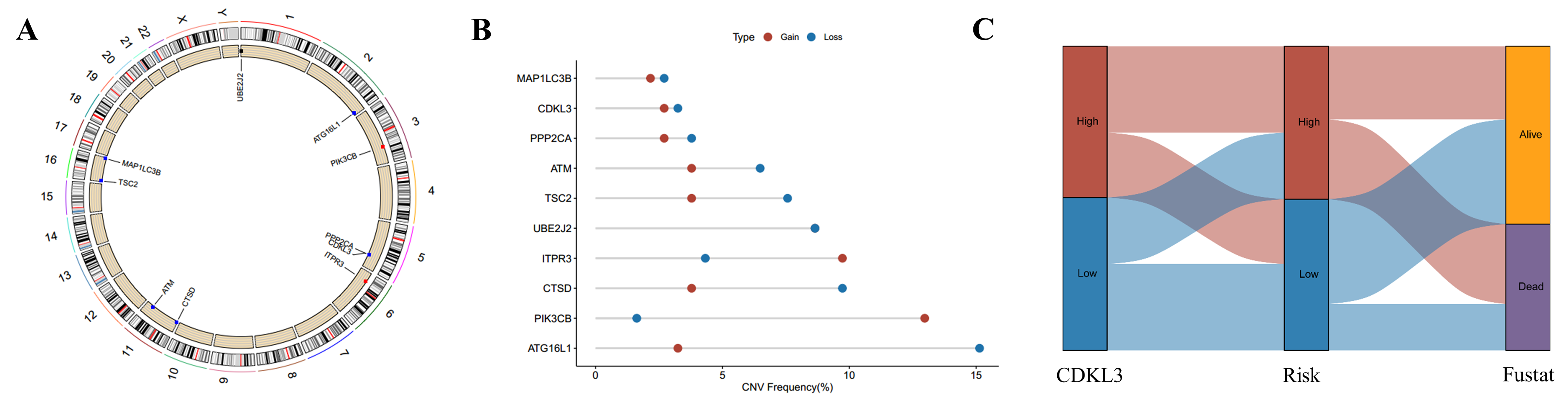

Supplement: Supplementary Figure 4 — The landscape in CDKL3 and the model genes in ESCA. (A) Circos plot of chromosomal distribution of CDKL3 and model genes. (B) CNV frequency of CDKL3 and model genes. The horizontal axis represents the change in frequency. (C) Sankey diagram of the relationship between CDKL3 group, risk group, and survival status. [file Image_4.tif]
